# Extraction of Cellulose Polymeric Material from *Populus tremula* Fibers: Characterization and Application to the Adsorption of Methylene Blue and Crystal Violet

**DOI:** 10.3390/polym13193334

**Published:** 2021-09-29

**Authors:** Faisal Muteb Almutairi, Yassine El-Ghoul, Mahjoub Jabli

**Affiliations:** 1 College of Science, Al-Imam Muhammad Ibn Saud Islamic University, Riyadh 11623, Saudi Arabia; 381110204@qu.edu.sa; 2 Department of Chemistry, College of Science, Qassim University, Buraidah 51452, Saudi Arabia; 3 Textile Engineering Laboratory, University of Monastir, Monastir 5019, Tunisia; 4 Department of Chemistry, College of Science Al-Zulfi, Majmaah University, Al-Majmaah 11952, Saudi Arabia; m.jabli@mu.edu.sa; 5 Textile Materials and Processes Research Unit, Tunisia National Engineering School of Monastir, University of Monastir, Monastir 5019, Tunisia

**Keywords:** cellulose, *Populus tremula*, FT-IR, SEM, XRD, TGA-DTA, methylene blue

## Abstract

Cellulose is the most widely available biopolymer which is extensively used for several applications including textiles, composites, pharmaceutical, water treatment, etc. In this investigation, cellulose was chemically extracted from *Populus tremula* seed fibers. Samples were characterized using FT-IR, SEM, XRD, and TGA-DTA analyses. FT-IR spectrum of the extracted cellulose confirmed that hemicellulose and lignin were removed during alkali and bleaching treatments. SEM images showed the partially roughened surface of the fiber due to the removal of non-cellulosic elements and surface impurities during chemical modification. The crystallinity index values for untreated *Populus tremula* fibers and extracted cellulose were calculated to be 32.8% and 58.9%, respectively. The obvious increase in the crystallinity index for the extracted cellulose confirmed the removal of amorphous compounds present in raw *populus*. Alkali-treated *populus* fibers were more thermally stable than raw fibers. All changes observed after alkali and bleaching treatments evidenced the removal of amorphous contents and non-cellulosic components in raw *populus* fibers. Extracted cellulose exhibited excellent adsorption capacities of methylene blue (140.4 mg g^−1^) and crystal violet (154 mg g^−1^). The pseudo second order equation fitted well the kinetic data indicating a chemi-sorption process. The Freundlich model complied well with the experimental data suggesting that the adsorption of the studied dyes was multilayer.

## 1. Introduction

Agricultural coproducts and by-products could be treated for the production of various materials (polymers, biofuels, chemicals, etc.). Most of biomass residues are lignocellulosic matters and cellulose is the main component [1,2,3]. Cellulose is the most widely available biopolymer which is extensively used for various applications including textiles, composites, pharmaceutical, energy, etc. [4,5,6,7].

Efforts have been made to produce natural cellulose fibers from biomass residues [8] covering several parts such as stems, leaves, roots, seeds, fruit, husks, etc. In this sense, many plants are investigated. Many research studies reported the multiple characteristics of natural fibers such as Prosopis juliflora [9], Indian areca fruit husk fibers [10], Saharan aloe vera cactus leaves [11], Citrullus lanatus climber [12], Vacheilla nilotica ssp indica tree [13], and so on. The major constituent of these natural fibers is cellulose, the reinforcing agent that provides the strength to these materials [14,15]. Cellulose is the major constituent of these natural extracted fibers, a reinforcing element which enables rigidity and strength to the plant. The other constituents are mainly hemicellulose and lignin which are more amorphous than cellulose. These branched polymers provide stiffness and compactness to the plant cell walls. Different parts of the plants contain these polymeric constituents of the natural fibers such as leaves, fruits, barks, bast, stalks, and in woods, etc. [16,17]. Among the recent fields of exploitation of these natural materials, we cite the treatment of contaminated waters via the adsorption process which is considered as simple, economic, and effective [18,19,20,21,22]. In line with this topic of valorization of natural extracted fibers, we have studied and characterized cellulosic fibers from nerium oleander [23], *Populus tremula* [24], and pergularia tomentosa [25] and we have investigated their application as bio-sorbents for various dyes from aqueous synthetic solutions. The different results of adsorption were promising depending on the chemical, physical, and structural properties of the extracted fibers.

*Populus tremula* has the biggest native range of any species from populus genus, being one of the most widely dispersed trees [26]. The literature revealed that only few works were focalized on the exploration of populus. For example, the study of Sezgin et al. [27] reported the kraft cooking of pinus pinaster and *Populus tremula* chip mixtures and the resulting pulp and paper properties were investigated. Marzena et al. [28] investigated the effects of *Populus tremula* hybridization with populus tremuloides michaux and populus alba L. on the growth and cellulosic pulp properties for papermaking applications. Rooni et al. [29] have pretreated *Populus tremula* with steam explosion and nitrogen explosive decompression to separate enzymatic hydrolysis and to increase bioethanol and biogas yields. To our knowledge, populus seed fibers is not explored for cellulose production. Herein, the aim of the current study was to extract a natural cellulosic fiber from *Populus tremula* seed plant. Afterward, the cellulosic extract was fully characterized using different chemical, structural, thermal, and morphological characterizations (FTIR, XRD, TGA-DTA, and SEM). The extracted cellulose was then applied as adsorbent of cationic dyes, methylene blue and crystal violet. The adsorption efficiency was evaluated by varying different experimental parameters such as pH, time, temperature, and the initial dye concentration. The theoretical kinetic and isothermal equations were investigated for the analysis of the evaluated experimental data.

## 2. Experimental Procedures

### 2.1. Materials and Reagents

*Populus tremula* seed fibers were collected from the region of Bizerte (Tunisia) during the summer (July). Glacial acetic acid and sodium hydroxide were used as laboratory grade reagents with pure quality. H_2_O_2_ (30%) was used to bleach the raw fibers as an oxidizing agent. Methylene blue (M.W = 319.85 g mol^−1^, λ _max_ in water = 665 nm, empirical formula: C_16_H_18_ClN_3_S) and crystal violet (M.W = 407.97 g mol^−1^, λ _max_ in water = 590 nm, empirical formula: C_25_N_3_H_30_Cl) were purchased from Sigma Aldrich company. Methylene blue and crystal violet solutions were prepared using distilled water.

### 2.2. Extraction of Cellulose

The extraction of cellulose from *Populus tremula* seed fibers was performed with reference to a method reported by Aiqin et al. [30] with a slight modification. First, the collected fibers were thoroughly washed with water to eliminate the sand and other vegetable impurities which are present on the surface. Then, they were spread on laboratory bench at room temperature until complete drying. After, the fibers were impregnated in a solution of 5 wt.% NaOH with a liquor ratio of 1:50 (*w*/*v*) at 80 °C for 2 h, in order to remove the wax and maximum of lignin. To remove the residual lignin and bleach the obtained organic matter, the alkali treated fibers were treated in a mixed solution of CH_3_COOH and H_2_O_2_ (*v*/*v* = 1:1) at 90 °C for 2 h with a liquor ratio of 1:50 (*w*/*v*). Finally, the resulting product was filtered using a Whatman filter paper and oven-dried at 60 °C for 6 h.

### 2.3. Characterization Instruments

The chemical structures of the studied samples were studied by means of an InfraLum FT-08 apparatus equipped with ATR (Serial number: 211157). The spectra were considered in 32 scans from 400 to 4000 cm^−1^ with a resolution of 4 cm^−1^. A JEOL JSM-5400 scanning electron microscope was used to examine the morphological characteristics of the studied samples. Gold was used to cover samples utilizing a vacuum sputter-coater to improve both conductivity and image quality with an accelerating voltage of 20 kV. A PANalytical X’Pert PRO MPD apparatus was employed to depict the XRD patterns in 2 theta range of 10°–90°. The samples were thermally analyzed in air flow, at a heating rate of 10°/min, in a Pt crucible with NETZSCH STA 449F3 instrument.

### 2.4. Adsorption Experiments

Adsorption experiments were conducted in batch mode using Erlenmeyer flasks holding 0.0125 g of extracted cellulose and a volume of 10 mL of methylene blue or crystal violet. The mixture was stirred at 125 rpm. After adsorption, the liquid was filtered and its absorbance was measured at the maximum wavelength (665 nm for methylene blue and 590 for crystal violet). The absorbance was checked at different period of times (0–120 min), methylene blue concentrations (0–1000 mg L^−1^), and temperature (22–55 °C). The adsorbed quantity of methylene blue or crystal violet was calculated using Equation (1):

(1) $$q\left(mg/g\right)=\frac{\left(C_{0}-C_{e}\right)\times V}{m}$$

where C_0_ and C_e_ represent the dye concentration at time = 0 min and at equilibrium, respectively. V is the volume of the dye used for experiment and m is the mass of the adsorbent.

## 3. Results and Discussion

### 3.1. FT-IR Spectroscopy Characterization

**Figure 1:**
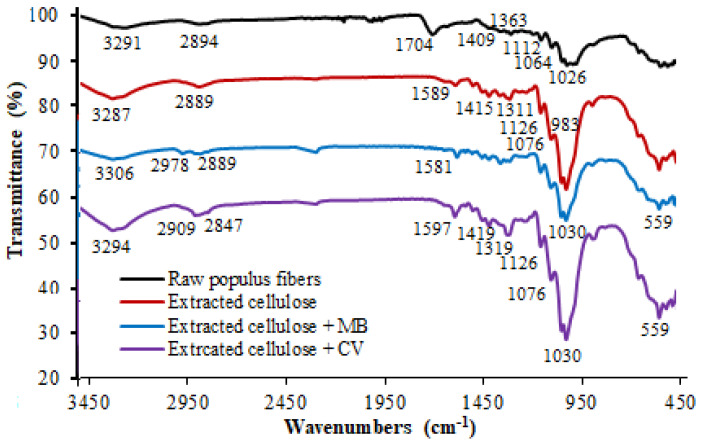
FT-IR spectrum of raw *Populus tremula* fibers and extracted cellulose before and after the adsorption of methylene blue and crystal violet (C_0_ = 30 mg L^−1^, Time = 30 min, T = 22 °C, dye volume = 10 mL, and adsorbent dosage = 0.012 g).

FT-IR spectra of raw *Populus tremula* fibers, extracted cellulose, MB adsorbed on extracted cellulose, and CV adsorbed on extracted cellulose are shown in Figure 1. FT-IR spectrum of the extracted cellulose confirmed that the hemicellulose and lignin were removed during alkali and bleaching treatments. The spectrum indicates that almost all the main absorption peaks of raw *populus* fibers are present in the spectrum of the extracted cellulose before and after dye adsorption. The absorption peak at 1704 cm^−1^ of raw *populus* fibers is attributed to C=O vibration of hemicellulose and lignin [31,32]. This peak disappears completely in the spectrum of extracted cellulose. The peak at around 3287–3306 cm^−1^, which corresponds to hydroxyl groups, is present in all spectra [30,31]. The peak at 2894 cm^−1^ corresponds to the aliphatic C–H vibration in cellulose [33,34,35,36]. The peaks observed at around 1368 cm^−1^ and 1064 cm^−1^ are attributed to C–O and C–H groups present in the cellulose polysaccharide ring and C–O–C in the pyranose ring, respectively. The high intensity peak at 983 cm^−1^ which is assigned to C–OH vibration confirms the presence of a high cellulose content [37,38]. For the spectra of the extracted cellulose carried out after the adsorption of methylene blue and crystal violet, there was no change in the main groups observed for cellulose structure. Only a small chemical shifting was noted for the absorption peaks related to OH, C–O–C, and C–OH groups. This suggests that the dyes molecules of the cationic dyes were interacted with cellulose via hydrogen bonding or electrostatic interactions.

### 3.2. SEM Images

**Figure 2:**
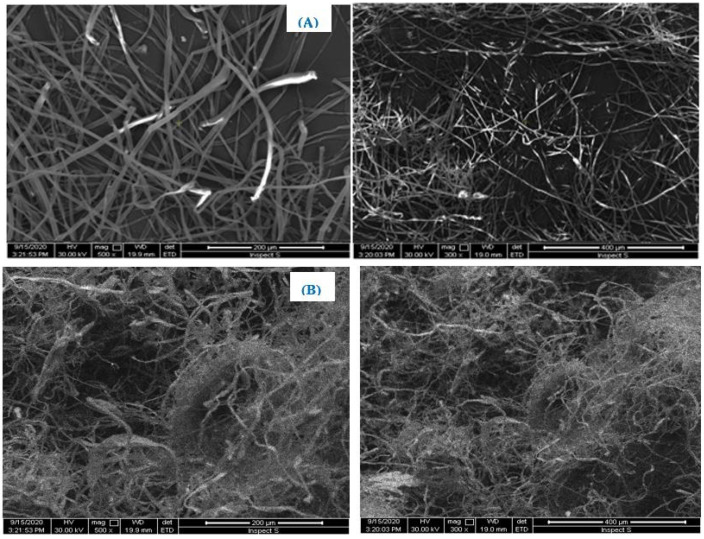
SEM images of: (**A**) untreated *Populus tremula* fibers and (**B**) extracted cellulose observed at different magnifications.

The raw and chemically treated *populus* fibers were morphologically investigated by SEM. The results indicated that the morphological characteristics are completely changed after chemical treatments. The raw fibers appeared individualized, twisted, and striated (Figure 2A). However, after alkali and bleaching treatments (Figure 2B), it is obvious that the organic matter became very clean and very condensed. This suggests that the non-cellulose materials and impurities from the surface of the fiber have been completely removed.

### 3.3. XRD Patterns

**Figure 3:**
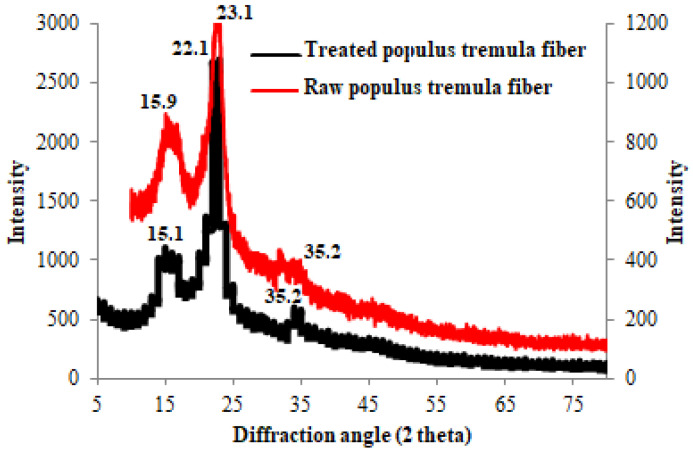
XRD patterns of untreated *Populus tremula* fibers and extracted cellulose.

Figure 3 gives the X-ray spectrum of untreated *Populus tremula* fibers and extracted cellulose. The diffraction patterns of the two samples show that cellulose of untreated and treated fibers is cellulose I as there is no doublet in intensity of the major crystalline peak [39,40]. The XRD spectrum or raw *populus* exhibits three peaks at 15.9°, 23.1°, and 35.2° whereas those for extracted cellulose are observed at 15.1°, 22.1°, and 35.2°. Indeed, these peaks correspond to (110), (200), and (040) lattice planes of crystalline cellulose I [41].

The crystallinity index (CrI) was calculated using Equation (2):

(2) $$\mathrm{CrI}\;\left(\%\right)=\frac{I_{200}-I_{am}}{I_{200}}\times 100$$

where *I*_200_ is the intensity of the highest crystalline peak and *I_am_* is the intensity of the predominantly amorphous peak.

The crystallinity index values for untreated *Populus tremula* fibers and extracted cellulose are calculated to be 32.8% and 58.9%, respectively. These calculated values, which are relatively low, indicated the presence of amorphous compounds including hemicelluloses and lignin in the composition of *populus* fiber [42,43]. The obvious increase of the crystallinity index for the extracted cellulose confirmed again the removal of amorphous compounds present in the raw fiber [44,45]. The obtained results are in line with those observed in the FT-IR interpretation.

### 3.4. Thermal Analyses: TGA/DTA

**Figure 4:**
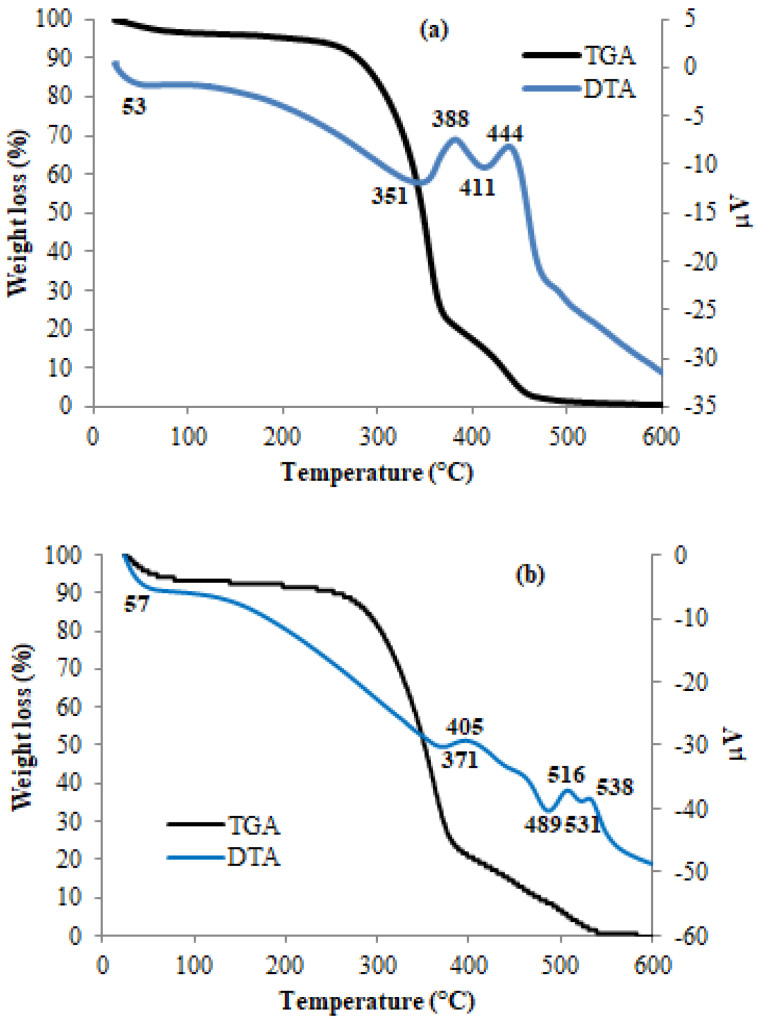
TGA/DTA curves of untreated *Populus tremula* fibers (**a**) and extracted cellulose (**b**) (Air flow, and heating rate of 10° min^−1^).

TGA is used to investigate the thermal stability of the studied materials. The TGA/DTA curves of untreated *Populus tremula* fibers and extracted cellulose are shown in Figure 4. The thermal decomposition of cellulose happens in different pyrolysis steps due to many reaction steps. At a lower temperature range, the studied samples have small weight losses (<10%) below 100 °C. This initial mass loss corresponds to moisture evaporation and absorbed water [46]. The total mass is reached at 500 °C and 550 °C for the untreated *Populus tremula* fibers and extracted cellulose, respectively. The DTA curve of the untreated *populus* fibers (Figure 4a) shows two exothermic peaks at 388°C and 444 °C which could be associated with the main pyrolytic reaction of the cellulose and the oxidation of the charred residues. However, after alkali and bleaching treatment of *populus* fibers (Figure 4b), the first exothermic peak is observed at higher temperature (405 °C) and two exotherms are seen at 516 °C and 538 °C. This thermal change indicates that the extracted cellulose is more thermally stable than the raw fiber. Such change in thermal event confirms again the chemical modification of *populus* fibers.

### 3.5. Application to the Adsorption of Methylene Blue and Crystal Violet

#### 3.5.1. Effect of Some Experimental Parameters on Adsorption

**Figure 5:**
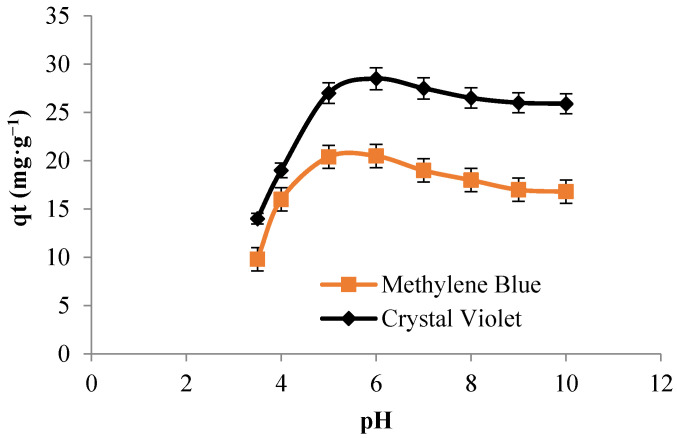
Effect of pH on the adsorption of: methylene blue and crystal violet (C_0_ = 30 mg L^−1^, Time = 30 min, T = 22 °C, Volume = 10 mL, and adsorbent dosage = 0.012 g) (For each measurement, 3 replicates are done).

The effect of the initial pH value on the adsorption of methylene blue and crystal violet is shown in Figure 5. The maximum of adsorption was reached at pH *=* 6. Indeed, at high acidic suspension, the protons H^+^ are present at high level causing therefore electrostatic repulsion with the cationic charges of the dye molecules. However, when the pH attains high values, the electrostatic forces of attraction between the positive charges of the cationic dyes and the negative charge of the surface of cellulose occurs, leading to an increase in the adsorption.

**Figure 6:**
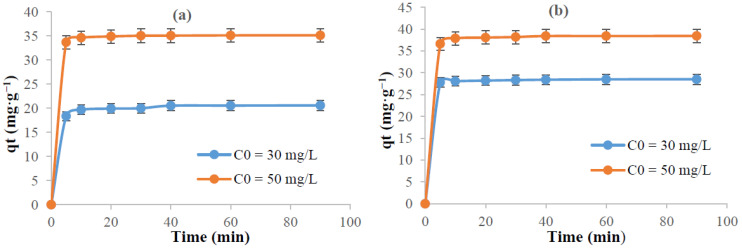
Effect of time on the adsorption of: (**a**) methylene blue and (**b**) crystal violet (pH = 6, T = 22°C, Volume = 10 mL, and adsorbent dosage = 0.012 g) (For each measurement, 3 replicates are done).

Figure 6 gives the progress of the adsorbed amount of dyes as a function of time. The results showed that the adsorption equilibrium was reached rapidly after 20 min of reaction. Two main stages characterize the adsorption profile kinetic curves. At the first 05 min, the adsorption rate occurs rapidly and about 90% of the target was achieved. After 10 min of reaction, the adsorption evolves slowly, and reaches its maximum at 20 min. This means that the active adsorption sites are saturated rapidly at this period of time.

**Figure 7:**
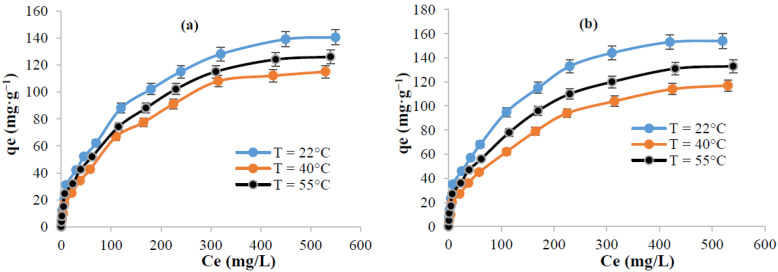
Effect of temperature on the adsorption of: (**a**) methylene blue and (**b**) crystal violet (pH = 6, time = 40 min, Volume = 10 mL, and adsorbent dosage = 0.012 g) (For each measurement, 3 replicates are done).

Figure 7 represents the change of the adsorbed amount of dyes versus the temperature and the variation in the initial dye concentration. The results indicate that at equilibrium, the extracted cellulose adsorbs 140.4 mg·g^−1^ and 154 mg·g^−1^ of methylene blue and crystal violet, respectively. This variation in the adsorbed amounts between the two cationic dyes could be explained based on the difference in their chemical structures and molecular weights. It is worth mentioning that the adsorbed amount of methylene blue, using the extracted cellulose from *Populus tremula*, is much higher than some previously reported adsorption capacities by applying, for example, orange peel (18.6 mg·g^−1^) [47], jute processing waste (22.47 mg·g^−1^) [48], plam tree waste (39.47 mg·g^−1^) [49], and rice husk (40.6 mg·g^−1^) [50]. It is comparable to some other studied biosorbants such as kenaf core fibers (131.6 mg·g^−1^) [51], mango seed kernel (142.9 mg·g^−1^) [52], and rejected tea (147 mg·g^−1^) [53]. The adsorption phenomenon followed an exothermic mode. As an example, at 55 °C, the adsorbed amount of methylene blue decreases from 140.4 mg·g^−1^ to 115 mg·g^−1^. This behavior could be explained by the decrease of the interaction between dye molecules and active sites present in cellulose surface revealing a phenomenon of desorption.

#### 3.5.2. Kinetic Study

**Figure 8:**
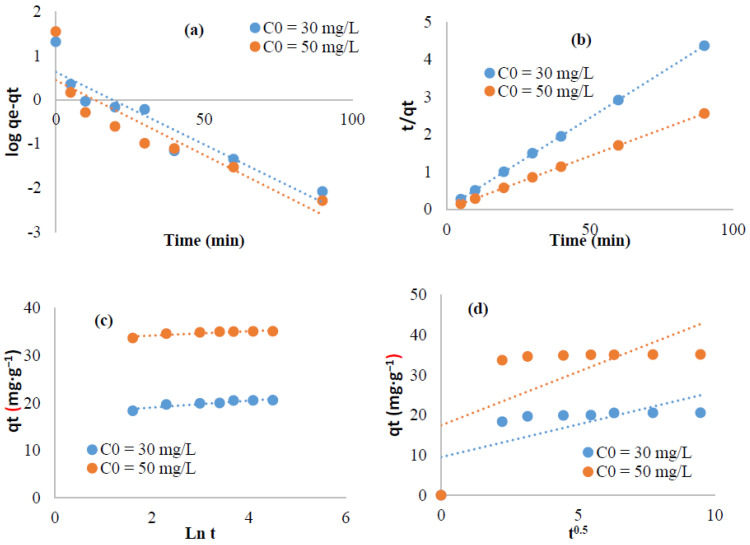
Linearized kinetic data for Methylene blue adsorption: (**a**) First pseudo order, (**b**) Pseudo second order, (**c**,**d**) Intra-particular diffusion.

**Figure 9:**
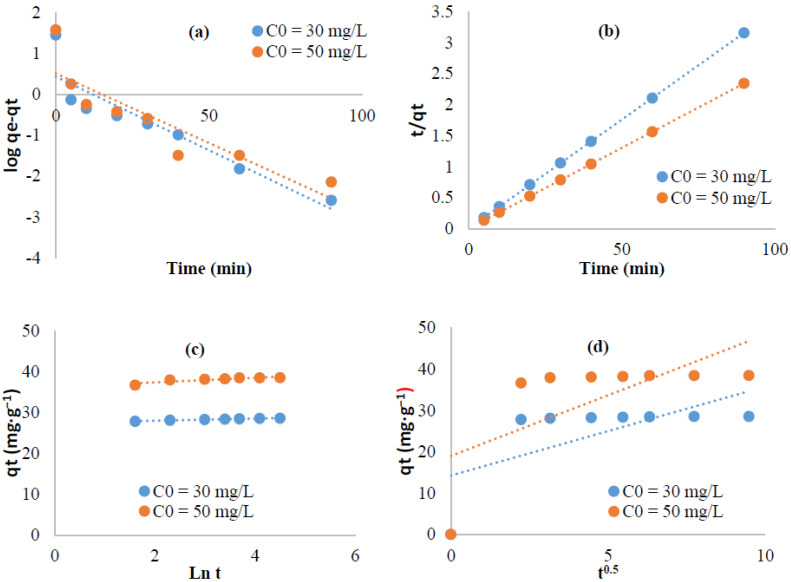
Linearized kinetic data for Crystal violet adsorption: (**a**) First pseudo order, (**b**) Pseudo second order, (**c**,**d**) Intra-particular diffusion.

The mechanism of the adsorption of methylene blue and crystal violet on the surface of the extracted cellulose as adsorbent is analyzed using pseudo first order, pseudo second order, Elovich, and intra-particular diffusion equations (Figure 8 and Figure 9). The computed parameters are summarized in Table 1 and Table 2. For the pseudo second order data, the high R^2^ values (R^2^ ≥ 0.99) and the excellent coincidence of the calculated adsorption capacities (q_th_) with those determined experimentally (q_exp_) strongly suggested convenience of this equation. This suggests that the second order equation fitted well our kinetic data indicating a chemi-sorption process [54,55]. The plots of q_t_ versus t^1/2^ (Figure 8d and Figure 9d) deviated from the origin which means that not only the intra-particular diffusion could control the adsorption phenomenon but also more than one kinetic process could occur [53].

#### 3.5.3. Isotherms Study and Thermodynamic Parameters Determination

**Figure 10:**
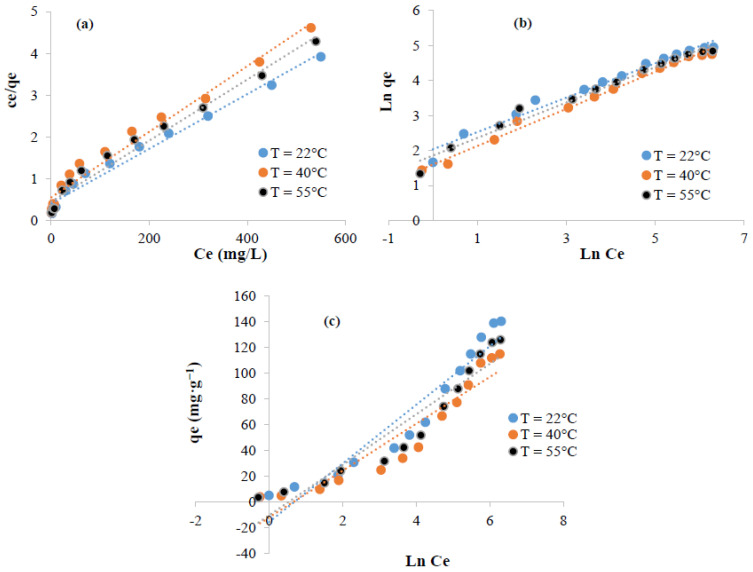
Linearized isotherms data for the adsorption of methylene blue on the surface of extracted cellulose: (**a**) Langmuir, (**b**) Freundlich, and (**c**) Temkin.

**Figure 11:**
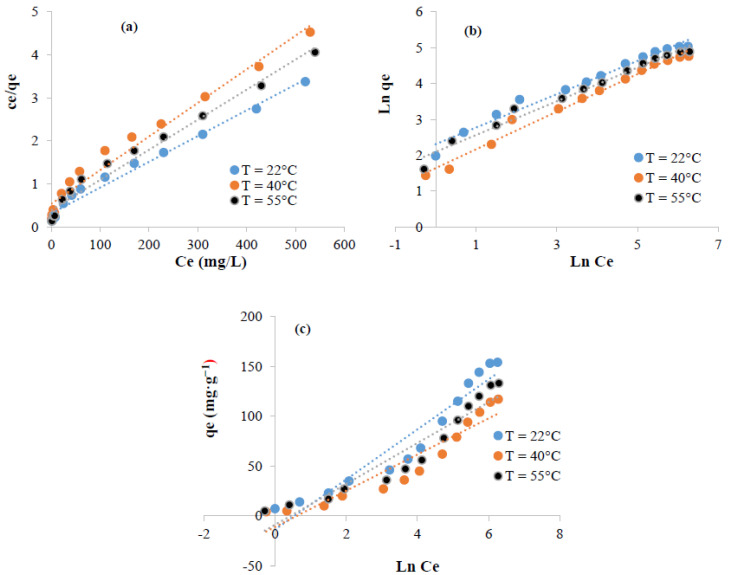
Linearized isotherms data for the adsorption of crystal violet on the surface of extracted cellulose: (**a**) Langmuir, (**b**) Freundlich, and (**c**) Temkin.

The adsorption of methylene blue and crystal violet was analyzed using Langmuir, Freundlich, and Temkin (Figure 10 and Figure 11). The fitting parameters are summarized in Table 1 and Table 2. The Freundlich model appeared more suitable to fit the experimental data (R^2^ ≥ 0.98) suggesting that the sorption of methylene blue and crystal violet was multilayer. The favorability of the phenomenon could be, also, judged using the parameter “*n*” computed from Freundlich model. From the data, 1 < *n* suggests that the sorption of these dye molecules was favorable [56,57].

**Figure 12:**
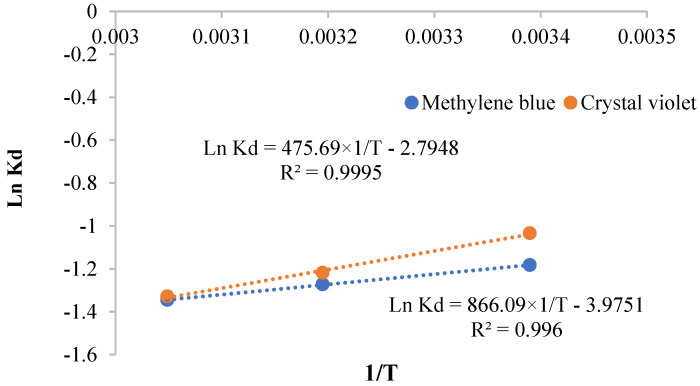
Change of Ln Kd versus 1/T for methylene blue and crystal violet.

The thermodynamic parameters ΔH° and ΔS° were calculated from the plots of Ln (K_L_) against 1/T (Figure 12). The negative value of ΔH° confirmed that the interaction between extracted cellulose and the cationic dyes is exothermic. This result is in line with the decrease of the adsorbed amounts of dyes with the raise in temperature. The negative values of ΔS° suggested the decrease of the disorder in the studied system adsorbate–adsorbent in which some structural changes could happen [58]. The positive values of ΔG° revealed that the adsorption of methylene blue and crystal violet is non-spontaneous.

## 4. Conclusions

To sum up the findings, cellulose was chemically extracted from *Populus tremula* seed fibers and analyzed using FT-IR, SEM, XRD, and TGA-DTA techniques. FT-IR results proved that the hemicellulose and lignin were removed during alkali and bleaching treatments. The crystallinity index values for untreated *Populus tremula* fibers and extracted cellulose were calculated to be 32.8% and 58.9%, respectively, confirming also the removal of amorphous compounds present in raw *populus*. TGA/DTA results indicated that the extracted cellulose was more thermally stable than raw fibers. Such observed events evidenced the removal of amorphous contents and non-cellulosic components in raw *populus* fibers after chemical treatments. The extracted cellulose was proved to be an excellent adsorbent of cationic dyes, i.e., methylene blue (140.4 mg·g^−1^) and crystal violet (154 mg·g^−1^). The high adsorbed amounts of dyes confirmed the efficiency of using the extracted cellulose to treat contaminated waters. The pseudo second order equation described well the kinetic data suggesting a chemi-sorption process. The Freundlich model fitted well the experimental data indicating that the adsorption of the cationic dyes was a multilayer one. The interaction between the extracted cellulose and cationic dyes is exothermic. The adsorption of methylene blue and crystal violet is non-spontaneous. Further investigations will be extended to assess the extracted cellulose for other purposes including composite material design and to chemically functionalize the resulting cellulose polymeric material with aminated reagents.

## Figures and Tables

**Table 1:** Summarized kinetic data, Langmuir, Freundlich, and Temkin parameters for the adsorption of methylene blue on the surface of extracted cellulose from *Populus tremula*.

Kinetic equation	Constants	Dye concentration		Isotherms	Parameters	Temperature		
Pseudo first order		30 mg L^−1^	50 mg L^−1^			22	40	55
	K_1_ (min^−1^)	0.0328	0.0339		q_m_ (mg·g^−1^)	153.84	126.58	136.98
	q (mg·g^−1^)	4.22	2.76	Langmuir	K_L_ (L·g^−1^)	0.016	0.014	0.016
	R^2^	0.88	0.8		R^2^	0.98	0.97	0.97
Pseudo second order	K_2_	0.068	0.155	Thermodynamic parameters	ΔH° (KJ mol^−1^)	−7.20		
	q	20.75	35.21		ΔS° (J mol^−1^)	−33.049		
	R^2^	0.99	1		ΔG° (KJ mol^−1^)	2.55	3.14	3.64
				Freundlich	K_F_ (L·g^−1^)	108.26	39.67	71.53
Elovich	*α* (mg·g^−1^·min^−1^)	2.68 × 10^10^	2.62 × 10^31^		*n*	2.036	1.88	1.98
	*β* (mg·g^−1^·min^−1^)	1.38	2.19		R^2^	0.98	0.99	0.98
	R^2^	0.86	0.79	Temkin	b_T_ (J.mol^−1^)	107.86	143.23	139.13
Intra-particular- Diffusion	K (mg·g^1^·min^1/2^)	1.63	2.66		A (L·g^−1^)	1.96	1.91	1.68
	R^2^	0.5	0.44		R^2^	0.92	0.89	0.91

**Table 2:** Summarized kinetic data, Langmuir, Freundlich, and Temkin parameters for the adsorption of crystal violet on the surface of extracted cellulose from *Populus tremula*.

Kinetic equation	Constants	Dye concentration		Isotherms	Parameters	Temperature		
Pseudo first order		30 mg L^−1^	50 mg L^−1^			22	40	55
	K_1_ (min^−1^)	0.0361	0.0341		q_m_ (mg·g^−1^)	166.66	128.20	142.85
	q (mg·g^−1^)	2.75	3.29	Langmuir	K_L_ (L·g^−1^)	0.019	0.014	0.017
	R^2^	0.85	0.79		R^2^	0.98	0.97	0.97
Pseudo second order	K_2_	0.036	0.129	Thermodynamic parameters	ΔH° (KJ mol^−1^)	−3.95		
	q	28.57	38.61		ΔS° (J mol^−1^)	−23.23		
	R^2^	1	1		ΔG° (KJ mol^−1^)	2.90	3.32	3.66
				Freundlich	K_F_ (L·g^−1^)	203.89	43.79	123.96
Elovich	*α* (mg·g^−1^·min^−1^)	4.75 × 10^45^	5.67 × 10^27^		*n*	2.15	1.90	2.12
	*β* (mg·g^−1^·min^−1^)	3.88	1.78		R^2^	0.98	0.99	0.98
	R^2^	0.97	0.79	Temkin	b_T_ (J.mol^−1^)	97.77	143.39	133.16
Intra-particular- Diffusion	K (mg·g^1^·min^1/^^2^)	2.145	2.925		A (L·g^−1^)	1.713	1.833	1.534
	R^2^	0.43	0.44		R^2^	0.92	0.90	0.91

## Data Availability

The data presented in this study are available on request from the corresponding author.
